# Extraction of new solitary wave solutions in a generalized nonlinear Schrödinger equation comprising weak nonlocality

**DOI:** 10.1371/journal.pone.0297898

**Published:** 2024-05-14

**Authors:** Miguel Vivas-Cortez, Ghada Ali Basendwah, Beenish Rani, Nauman Raza, Mohammed Kbiri Alaoui

**Affiliations:** 1 School of Physical and Mathematical Sciences, Faculty of Exact and Natural Sciences, Pontificia Universidad Catolica del Ecuador, Apartado, Quito, Ecuador; 2 Department of Mathematics, King Abdulaziz University, Jeddah, Saudi Arabia; 3 Department of Mathematics, University of the Punjab, Lahore, Pakistan; 4 Department of Mathematics, College of Science, King Khalid University, Abha, Saudi Arabia; Tel Aviv University, ISRAEL

## Abstract

This article delves into examining exact soliton solutions within the context of the generalized nonlinear Schrödinger equation. It covers higher-order dispersion with higher order nonlinearity and a parameter associated with weak nonlocality. To tackle this equation, two reputable methods are harnessed: the sine-Gordon expansion method and the $G^{\prime }/\left(bG^{\prime }+G+a\right)$-expansion method. These methods are employed alongside suitable traveling wave transformation to yield novel, efficient single-wave soliton solutions for the governing model. To deepen our grasp of the equation’s physical significance, we utilize Wolfram Mathematica 12, a computational tool, to produce both 3D and 2D visual depictions. These graphical representations shed light on diverse facets of the equation’s dynamics, offering invaluable insights. Through the manipulation of parameter values, we achieve an array of solutions, encompassing kink-type, dark soliton, and solitary wave solutions. Our computational analysis affirms the effectiveness and versatility of our methods in tackling a wide spectrum of nonlinear challenges within the domains of mathematical science and engineering.

## Introduction

Nonlinear phenomena manifesting in a fields such as engineering, physics, computational mathematics, chemistry, and biological sciences find precise representation and simulation through nonlinear evolution equations (NLEEs) [1]. The importance of nonlinearity in wave dynamics is unmistakable within the realm of nonlinear sciences. Recent years have witnessed a concerted research endeavor aimed at unraveling analytical solutions, particularly solitary wave solutions, for NLEEs. To efficiently solve NLEEs and provide insights into intricate procedures, researchers have developed a variety of tools, including computational algorithms, analytical and numerical techniques. Several methods for analyzing these data have been proposed, such as modified generalized Riccati equation mapping approach [2], Lie symmetry analysis method [3], Hirota’s method [4], inverse Fourier transform [5] and many others.

Solitons, also known as invariant or solitary wave solutions, are stable, non-dispersive wave events that preserve their shape and speed as they propagate. These solutions, which are present in many physical systems, including nonlinear optics and water waves, are distinguished by their capacity to hold their original shape even when subjected to particular changes. Notably, solitons usually do not show singularities, which adds to their importance in comprehending and simulating wave behavior. Many methods have been developed to find soliton solutions. Researchers have used a variety of mathematical methods, such as Kumar et al. [6] employed generalized exponential rational function technique to obtain the closed-form soliton solutions, Rani et al. [7] used Lie symmetry analysis to attain explicit solutions, Dhiman and Kumar [8] utilized symmetry reduction to analyze the dynamics of invariant solition, *exp*(−*ϕ*(*ξ*)) expansion method is applied by Mathanaranjan [9] to find periodic and dark solitons, Zhao et al. [10] used exponential function technique to obtain soliton solutions and many others [11–13].

Erwin Schrödinger introduced the Schrödinger equation in 1925, which was formally published in 1926, laying the cornerstone for his subsequent scientific pursuits. The nonlinear Schrödinger equation (NLSE) holds a prominent position among the remarkable nonlinear scenarios emerging from mathematical modeling. Numerous scientific domains, spanning mathematical finance, optical fibers [14], biology, fluid dynamics [15], quantum mechanics [16], plasma physics [17], quantum field theory [18], and the propagation of solitary waves in piezoelectric materials [19], to name a few, encompass significant applications for this equation. Diverse effective methodologies, including the Kudryashov method [20], the unified technique [21], the sine-Gordon expansion technique [22], the exponential method [23], and the extended trial equation method [24], have been extensively employed in the formulation of soliton solutions for nonlinear partial differential equations, with a particular emphasis on NLSEs. As cited in reference [25], an overarching NLSE encompasses both Kerr nonlinearity and fourth-order dispersion effects, exerting influence over the propagation of light waves.

$$\begin{array}{c} \iota \frac{\partial P}{\partial t}+\frac{1}{24}C_{1}\frac{\partial^{4}P}{\partial x^{4}}+C_{2}{\left|P\right|}^{2}P=0. \end{array}$$

Within this particular model, the variables *t* and *x* signify the temporal and spatial dimensions, respectively. Meanwhile, the symbol *P* is employed to denote the complex envelope of the electric field. The cubic nonlinearity coefficient is denoted by the parameter *C*_2_, and the coefficient accounting for fourth-order diffraction or dispersion is represented as *C*_1_. When dealing with the propagation of small wavelengths in optical transmission materials, it becomes notably important to factor in nonlocal effects. As elucidated in reference [25], the aforementioned model necessitates refinement through the incorporation of an additional component to account for the influence of weak nonlocality.

$$\begin{array}{c} \iota \frac{\partial P}{\partial t}+\frac{1}{24}C_{1}\frac{\partial^{4}P}{\partial x^{4}}+C_{2}{\left|P\right|}^{2}P+C_{3}P\frac{\partial^{2}{\left|P\right|}^{2}}{\partial x^{2}}=0. \end{array}$$ (1)

In this context, the term $\frac{\partial^{4}P}{\partial x^{4}}$ characterizes the impact of fourth-order dispersion, while |*P*|^2^*P* encapsulates the Kerr nonlinearity. Furthermore, the term *P*(|*P*|^2^)_*xx*_ signifies the presence of weak nonlinearity. Recently, this model has been employed for the investigation of optical pulse localization within guided wave structures, particularly in scenarios involving a quasi-periodic linear factor [26].

Our motivation, therefore, centers on the quest for soliton solutions within the aforementioned models. We intend to achieve this goal by leveraging recently established and reliable techniques, such as the sine-Gordon expansion technique and the $G^{\prime }/\left(bG^{\prime }+G+a\right)$-expansion method. These methods promise to address lingering questions from previous studies [27, 28]. The main aim of the sine-Gordon expansion method is to seek analytical solutions for a specific generalized nonlinear Schrödinger equation. Its core objective is to simplify the equation while preserving its fundamental characteristics. This standardization process paves the way for the discovery of exact solutions, thereby furnishing valuable insights into the behavior of the system described by the PDE [29]. In addition, we employ another method in this piece of research paper to obtain exact soliton solutions for a gNLSE through the $G^{\prime }/\left(bG^{\prime }+G+a\right)$-expansion method. This method offers a wide spectrum of solutions, encompassing singular exponential, trigonometric, and kink soliton solutions, as detailed in reference [30]. The discovered solitons have a crucial role in atmospheric research for controlling atmospheric gravity waves, maintaining wave stability, and preventing dispersion. Long-range coherent wave structure maintenance is essential for comprehending global atmospheric circulation patterns and how they affect weather occurrences. The effectiveness of the proposed methods in comparison to existing methods is determined by their problem-specific success and ability to provide accurate solutions, with factors such as ease of implementation, generality, and computational efficiency playing important roles in their evaluation. Consequently, our study employs mathematical computations and graphical analysis to scrutinize the physical characteristics associated with these waveform shapes [31].

The significance of this study, which includes weak nonlocality, lies in its ability to offer a robust mathematical framework that effectively captures the intricate interplay between nonlinearity and the subtle effects of weak nonlocality. This equation is particularly pertinent in the investigation of kink, dark, and novel solitary waves within nonlinear optical systems. Its value extends to serving as a valuable tool for comprehending and forecasting the behavior of solitary waves across diverse physical and optical scenarios.

The paper’s structure unfolds as follows: In Section 2, we embark on a mathematical scrutiny of the model, aiming to derive the ordinary differential equation (ODE). Sections 3 and 4 delve into an extensive exploration and practical application of the sine-Gordon expansion method. Moving forward, Sections 5 and 6 shed light on the fundamental elements and practical utility of the $G^{\prime }/\left(bG^{\prime }+G+a\right)$-expansion method. Section 7 presents a comprehensive overview of graphical illustrations, enriching our grasp of the subject matter. Lastly, Section 8 encapsulates our findings and contributions, serving as a concluding section that distills the pivotal insights of this study. Moreover, we also discuss future directions.

## Section 2: Mathematical analysis

For crafting solutions within this specific model, we employ the following Ansatz:

$$\begin{array}{c} P\left(x,t\right)=p\left(\zeta \right)e^{\iota \beta t},\;\zeta =\alpha x. \end{array}$$ (2)

Swapping the expression from Eq (2) into Eq (1) generates the following ODE:

$$\begin{array}{c} C_{1}\alpha^{4}\frac{d^{4}p\left(\zeta \right)}{d\zeta^{4}}+48C_{3}\alpha^{2}p^{2}\left(\zeta \right)\frac{d^{2}p\left(\zeta \right)}{d\zeta^{2}}+48C_{3}\alpha^{2}p\left(\zeta \right){\left(\frac{dp(\zeta )}{d\zeta }\right)}^{2}-24\beta p\left(\zeta \right)+24C_{2}p^{3}\left(\zeta \right)=0.\;\; \end{array}$$ (3)

## Section 3: Analysis of the sine-Gordon expansion method

Contemplating the sine-Gordon equation presented below:

$$\begin{array}{c} P_{xx}-P_{tt}=e^{2}\;\text{sin}\left(P\right), \end{array}$$ (4)

Here, we have the function denoted as *P*(*x*, *t*), and ‘e’ signifies any non-zero real number.

**Step 1:** Upon employing the traveling wave transformation *P*(*x*, *t*) = *p*(*ζ*), where *ζ* = *x* − *Ξt*, Eq (4) simplifies into the subsequent nonlinear ODE.

$$\begin{array}{c} p^{\prime \prime }=\frac{e^{2}}{1-\Xi^{2}}\;\text{sin}\left(p\right) \end{array}$$ (5)

While considering the function as *P* defined by *p*(*ζ*), where *ζ* represents the amplitude of the traveling wave, and *Ξ* signifies the velocity of wave propagation, integration of the aforementioned equation results in the following expression:

$$\begin{array}{c} {\left[{\left(\frac{p}{2}\right)}^{\prime }\right]}^{2}=\frac{e^{2}}{1-\Xi^{2}}\;{\text{sin}}^{2}(\frac{p}{2})+B. \end{array}$$ (6)

Here, *B* represents the constant of integration.

Taking *B* = 0, $\frac{p}{2}=\vartheta \left(\zeta \right)$ and $h^{2}=\frac{e^{2}}{1-\Xi^{2}}$.

Eq (6) assumes the subsequent form.

$$\begin{array}{c} \vartheta^{\prime }=h\;\text{sin}\left(\vartheta \right). \end{array}$$ (7)

When we opt for *h* = 1, the equation above simplifies to the following form.

$$\begin{array}{c} \vartheta^{\prime }=\;\text{sin}\left(\vartheta \right). \end{array}$$ (8)

Applying the separation of variables method to solve Eq (8), we derive two significant relationships as follows:

$$\begin{array}{c} \;\text{sin}\;\vartheta =\;\text{sin}\left(\vartheta \left(\zeta \right)\right)=\frac{2me^{\zeta }}{m^{2}e^{2\zeta }+1}{|}_{m=1}=\left(\zeta \right), \end{array}$$ (9)

$$\begin{array}{c} \text{cos}\;\vartheta =\;\text{cos}\left(\vartheta \left(\zeta \right)\right)=\frac{m^{2}e^{2\zeta }-1}{m^{2}e^{2\zeta }+1}{|}_{m=1}=\text{tanh}\left(\zeta \right), \end{array}$$ (10)

*m* ≠ 0 is the integrating constant.

**Step 2.** The anticipated solution for Eq (5) can be expressed as follows.

$$\begin{array}{c} p\left(\zeta \right)=\sum_{i=1}^{ℵ}\;{\text{tanh}}^{j-1}\left(\zeta \right)[B_{j}\left(\zeta \right)+A_{j}\;\text{tanh}\left(\zeta \right)]+A_{0}. \end{array}$$ (11)

Using Eqs (9) and (10), the above Eq (11) becomes

$$\begin{array}{c} p\left(\vartheta \right)=\sum_{j=1}^{ℵ}{\;\text{cos}}^{\;j-1}\left(\vartheta \right)[B_{j}\;\text{sin}\left(\vartheta \right)+A_{j}\;\text{cos}\left(\vartheta \right)]+A_{0}. \end{array}$$ (12)

To determine the positive integer ℵ, we utilize the balancing principle, a method that considers the balance between the highest-order derivative and the most influential nonlinear term found within the nonlinear ODE.

**Step 4:**

Upon inserting Eq (12) into the transformed ODE, we arrive at an algebraic equation comprising various powers of sin(*ϑ*)cos(*ϑ*). By setting the coefficients of each power to zero, we establish a system of algebraic equations featuring the variables *A*_0_, *A*_*j*_, and *B*_*j*_. By solving this resulting system for *A*_0_, *A*_*j*_, and *B*_*j*_ and subsequently substituting these values into Eq (11), we can construct a range of exact solutions for the transformed ODE.

## Section 4: Applications of the sine-Gordon expansion method

In this section, we employ the sine-Gordon expansion method (SGEM) to solve Eq (3) and derive soliton solutions. Once we apply the balancing procedure, the solution takes the following form.

$$\begin{array}{c} p\left(\vartheta \right)=B_{1}\;\text{sin}\left(\vartheta \right)+A_{1}\;\text{cos}\left(\vartheta \right)+A_{0}. \end{array}$$ (13)

Employing the solution methodology as elucidated in Section 3, we calculate the values of the unknowns *A*_0_, *A*_1_, and *B*_1_, yielding the following results.

**SET 1:**

$$\begin{array}{ccc} & & A_{0}=0,\;A_{1}=-\frac{\sqrt{-C_{1}}\sqrt{C_{2}}}{6C_{3}},\;B_{1}=0,\;\beta =\frac{-C_{1}C_{2}^{2}}{36C_{3}^{2}},\;\alpha =-\frac{\sqrt{C_{2}}}{\sqrt{2}C_{3}}. \end{array}$$

By utilizing the values of *A*_0_, *A*_1_, *B*_1_, *β*, and *α* obtained from **SET 1** in Eq (13), and subsequently substituting them into Eq (2), we observe the solution as follows:

$$\begin{array}{c} P_{1}\left(x,t\right)=-\frac{\sqrt{-C_{1}}\sqrt{C_{2}}}{6C_{3}}\;\text{tanh}\left(-\frac{\sqrt{C_{2}}}{\sqrt{2}C_{3}}x\right)e^{-\frac{i\text{C1}{\text{C2}}^{2}t}{36{\text{C3}}^{2}}}. \end{array}$$

**SET 2:**

$$\begin{array}{ccc} & & A_{0}=0,\;A_{1}=\frac{\sqrt{-C_{1}}\sqrt{C_{2}}}{6C_{3}},\;B_{1}=0,\;\beta =\frac{-C_{1}C_{2}^{2}}{36C_{3}^{2}},\;\alpha =-\frac{\sqrt{C_{2}}}{\sqrt{2}C_{3}}. \end{array}$$

Similarly, when we employ the values of *A*_0_, *A*_1_, *B*_1_, *β*, and *α* from **SET 2** in Eq (13), and subsequently substitute them into Eq (2), the solution appears as follows.

$$\begin{array}{c} P_{2}\left(x,t\right)=\frac{\sqrt{-C_{1}}\sqrt{C_{2}}}{6C_{3}}\;\text{tanh}\left(-\frac{\sqrt{C_{2}}}{\sqrt{2}C_{3}}x\right)e^{-\frac{i\text{C1}{\text{C2}}^{2}t}{36{\text{C3}}^{2}}}. \end{array}$$

## Section 5:The $G^{\prime }/\left(bG^{\prime }+G+a\right)$-expansion method

Suppose that the transformed ODE possesses a solution in the following manner.

$$\begin{array}{c} P\left(\zeta \right)=\sum_{j=0}^{ℵ}F_{j}V^{j}, \end{array}$$ (14)

Here, we have $V=V\left(\zeta \right)=\frac{G^{\prime }}{bG^{\prime }+G+a}$, with a and b being nonzero parameters. The value of ℵ can be ascertained using the principle of homogeneous balancing. Additionally, we introduce arbitrary constants denoted as *F*_*j*_, which will be determined subsequently. Furthermore, *V* = *V*(*ζ*) satisfies the subsequent ODE.

$$\begin{array}{c} V^{\prime }=\frac{dV(\zeta )}{d\zeta }=\left(\lambda -\mu -1\right)V^{2}+\frac{2\mu -\lambda }{b}V-\frac{\mu }{b^{2}}. \end{array}$$ (15)

Additionally, we have *G* = *G*(*ζ*) which represents the solution of the following ODE.

$$\begin{array}{c} G^{\prime \prime }=-\frac{\lambda }{b}G^{\prime }-\frac{\mu }{b^{2}}G-\frac{\mu }{b^{2}}a, \end{array}$$ (16)

In Eq (15), we encounter real numbers λ and *μ*. This equation exhibits the following categories of solutions,

**Type 1**: If Θ = λ^2^ − 4*μ* > 0, then $G=-a+r_{1}e^{\frac{1}{2b}\left(-\lambda -\sqrt{\Theta }\right)\zeta }+r_{2}e^{\frac{1}{2b}\left(-\lambda +\sqrt{\Theta }\right)\zeta }$, *r*_1_ and *r*_2_ are arbitrary constants satisfying $a^{2}+r_{1}^{2}+r_{2}^{2}\neq 0$.

In this type, *V* = *V*(*ζ*) is represented as follows:

$$\begin{array}{c} V=\frac{r_{1}\left(\lambda +\sqrt{\Theta }\right)+r_{2}\left(\lambda -\sqrt{\Theta }\right)e^{\frac{\sqrt{\Theta }}{b}\zeta }}{b\;r_{1}\left(\lambda -2+\sqrt{\Theta }\right)+b\;r_{2}\left(\lambda -2-\sqrt{\Theta }\right)e^{\frac{\sqrt{\Theta }}{b}\zeta }}. \end{array}$$

We can alternatively express *V* = *V*(*ζ*) as follows:

$$\begin{array}{c} V=\frac{(\lambda \left(r_{2}-r_{1}\right)-\sqrt{\Theta }\left(r_{2}+r_{1}\right))\text{sinh}\left(\frac{\sqrt{\Theta }}{2b}\zeta \right)+(\lambda \left(r_{2}+r_{1}\right)-\sqrt{\Theta }\left(r_{2}-r_{1}\right))\text{cosh}\left(\frac{\sqrt{\Theta }}{2b}\zeta \right)}{b(\left(\lambda -2\right)\left(r_{2}-r_{1}\right)-\sqrt{\Theta }\left(r_{2}+r_{1}\right))\text{sinh}\left(\frac{\sqrt{\Theta }}{2b}\zeta \right)+b(\left(\lambda -2\right)\left(r_{2}+r_{1}\right)-\sqrt{\Theta }\left(r_{2}-r_{1}\right))\text{cosh}\left(\frac{\sqrt{\Theta }}{2b}\zeta \right)}. \end{array}$$

If $\left(\lambda -2\right)\left(r_{2}-r_{1}\right)-\sqrt{\Theta }\left(r_{2}+r_{1}\right)=0$, then

$$\begin{array}{c} V=\frac{\lambda -2\mu }{2b(\lambda -\mu -1)}-\frac{\sqrt{\Theta }}{2b(\lambda -\mu -1)}\;\text{tanh}\left(\frac{\sqrt{\Theta }}{2b}\zeta \right). \end{array}$$

If $\left(\lambda -2\right)\left(r_{2}+r_{1}\right)-\sqrt{\Theta }\left(r_{2}-r_{1}\right)=0$, then

$$\begin{array}{c} V=\frac{\lambda -2\mu }{2b(\lambda -\mu -1)}-\frac{\sqrt{\Theta }}{2b(\lambda -\mu -1)}\;\text{coth}\left(\frac{\sqrt{\Theta }}{2b}\zeta \right). \end{array}$$

**Type 2**: If Θ = λ^2^ − 4*μ* < 0, then $G=-a+e^{\frac{-\lambda }{2b}\zeta }(r_{1}\;\text{cos}\left(\frac{\sqrt{-\Theta }}{2b}\zeta \right)+r_{2}\;\text{sin}\left(\frac{\sqrt{-\Theta }}{2b}\zeta \right))$.

In this type 2, *V* = *V*(*ζ*) is represented in the following manner:

$$\begin{array}{c} V=\frac{(\lambda r_{1}-\sqrt{-\Theta }r_{2})\;\text{cos}\left(\frac{\sqrt{-\Theta }}{2b}\zeta \right)+(\lambda r_{2}-\sqrt{-\Theta }r_{1})\;\text{sin}\left(\frac{\sqrt{-\Theta }}{2b}\zeta \right)}{b(\left(\lambda -2\right)r_{1}-\sqrt{-\Theta }r_{2})\;\text{cos}\left(\frac{\sqrt{-\Theta }}{2b}\zeta \right)+b(\left(\lambda -2\right)r_{2}+\sqrt{-\Theta }r_{1})\;\text{sin}\left(\frac{\sqrt{-\Theta }}{2b}\zeta \right)}. \end{array}$$

If $\left(\lambda -2\right)r_{2}+\sqrt{-\Theta }r_{1}=0$, then

$$\begin{array}{c} V=\frac{\lambda -2\mu }{2b(\lambda -\mu -1)}+\frac{\sqrt{-\Theta }}{2b(\lambda -\mu -1)}\;\text{tan}\left(\frac{\sqrt{-\Theta }}{2b}\xi \right). \end{array}$$

If $\left(\lambda -2\right)r_{1}-\sqrt{-\Theta }r_{2}=0$, then

$$\begin{array}{c} V=\frac{\lambda -2\mu }{2b(\lambda -\mu -1)}-\frac{\sqrt{-\Theta }}{2b(\lambda -\mu -1)}\;\text{cot}\left(\frac{\sqrt{-\Theta }}{2b}\xi \right). \end{array}$$

By inserting Eqs (14) and (15) into the transformed ODE and setting the coefficients of *V*^*j*^ to zero, we establish a system of equations. Solving this resultant system of equations enables us to ascertain the values of the arbitrary constants.

## Section 6: Application of the $G^{\prime }/\left(bG^{\prime }+G+a\right)$-expansion method

In this subsection, we employ the $G^{\prime }/\left(bG^{\prime }+G+a\right)$-expansion method to extract soliton solutions from Eq (14). Following this method, for the case when *N* = 1, the assumed solution for Eq (14) takes the subsequent form.

$$\begin{array}{c} P\left(\xi \right)=F_{0}+F_{1}V, \end{array}$$ (17)

Here, we have the unknown constants represented as *F*_0_ and *F*_1_. Employing the $G^{\prime }/\left(bG^{\prime }+G+a\right)$-expansion method results in the following set of solutions.

**SET 3**

$$\begin{array}{c} F_{0}=\frac{\sqrt{C_{1}}\sqrt{C_{2}}\left(\lambda -2\mu \right)}{2\sqrt{3C_{3}^{2}(\lambda^{2}-4\mu )}},F_{1}=\frac{b\sqrt{C_{1}}\sqrt{C_{2}}\left(-\lambda +\mu +1\right)}{\sqrt{3}\sqrt{C_{3}^{2}(\lambda^{2}-4\mu )}},\alpha =-\frac{\sqrt{2}b\sqrt{C_{2}}}{\sqrt{C_{3}(-(\lambda^{2}-4\mu ))}},\beta =\frac{C_{1}C_{2}^{2}}{12C_{3}^{2}}. \end{array}$$

The solutions corresponding to **SET 3** are presented below. In accordance with **Type 1**, when Θ = λ^2^ − 4*μ* > 0, we obtain the following results

$$\begin{array}{c} P_{3}\left(x,t\right)=(\frac{\sqrt{C_{1}}\sqrt{C_{2}}\left(\lambda -2\mu \right)}{2\sqrt{3C_{3}^{2}(\lambda^{2}-4\mu )}}+\frac{b\sqrt{C_{1}}\sqrt{C_{2}}\left(-\lambda +\mu +1\right)}{\sqrt{3}\sqrt{C_{3}^{2}(\lambda^{2}-4\mu )}}V)e^{\left(\frac{itC_{1}C_{2}^{2}}{12C_{3}^{2}}\right)}, \end{array}$$ (18)

where,

$$\begin{array}{c} V=\frac{(\lambda \left(r_{2}-r_{1}\right)-\sqrt{\Theta }\left(r_{2}+r_{1}\right))\text{sinh}\left(\frac{\sqrt{\Theta }}{2b}\xi \right)+(\lambda \left(r_{2}+r_{1}\right)-\sqrt{\Theta }\left(r_{2}-r_{1}\right))\text{cosh}\left(\frac{\sqrt{\Theta }}{2b}\xi \right)}{b(\left(\lambda -2\right)\left(r_{2}-r_{1}\right)-\sqrt{\Theta }\left(r_{2}+r_{1}\right))\text{sinh}\left(\frac{\sqrt{\Theta }}{2b}\xi \right)+b(\left(\lambda -2\right)\left(r_{2}+r_{1}\right)-\sqrt{\Theta }\left(r_{2}-r_{1}\right))\text{cosh}\left(\frac{\sqrt{\Theta }}{2b}\xi \right)}. \end{array}$$

Moreover, if $\left(\lambda -2\right)\left(r_{2}-r_{1}\right)-\sqrt{\Theta }\left(r_{2}+r_{1}\right)=0$, then Eq (18) becomes

$$\begin{array}{c} P_{4}\left(x,t\right)=(\frac{\sqrt{C_{1}}\sqrt{C_{2}}\left(\lambda -2\mu \right)}{2\sqrt{3C_{3}^{2}(\lambda^{2}-4\mu )}}+\frac{b\sqrt{C_{1}}\sqrt{C_{2}}\left(-\lambda +\mu +1\right)}{\sqrt{3}\sqrt{C_{3}^{2}(\lambda^{2}-4\mu )}}V)e^{\left(\frac{itC_{1}C_{2}^{2}}{12C_{3}^{2}}\right)}, \end{array}$$ (19)

where,

$$\begin{array}{c} V=\frac{\lambda -2\mu }{2b(\lambda -\mu -1)}-\frac{\sqrt{\Theta }}{2b(\lambda -\mu -1)}\;\text{tanh}\left(\frac{\sqrt{\Theta }}{2b}\xi \right). \end{array}$$

Moreover, if $\left(\lambda -2\right)\left(r_{2}+r_{1}\right)-\sqrt{\Theta }\left(r_{2}-r_{1}\right)=0$, then Eq (18) becomes

$$\begin{array}{c} P_{5}\left(x,t\right)=(\frac{\sqrt{C_{1}}\sqrt{C_{2}}\left(\lambda -2\mu \right)}{2\sqrt{3C_{3}^{2}(\lambda^{2}-4\mu )}}+\frac{b\sqrt{C_{1}}\sqrt{C_{2}}\left(-\lambda +\mu +1\right)}{\sqrt{3}\sqrt{C_{3}^{2}(\lambda^{2}-4\mu )}}V)e^{\left(\frac{itC_{1}C_{2}^{2}}{12C_{3}^{2}}\right)}, \end{array}$$ (20)

where,

$$\begin{array}{c} V=\frac{\lambda -2\mu }{2b(\lambda -\mu -1)}-\frac{\sqrt{\Theta }}{2b(\lambda -\mu -1)}\;\text{coth}\left(\frac{\sqrt{\Theta }}{2b}\xi \right). \end{array}$$

In alignment with **Type 2**, when Θ = λ^2^ − 4*μ* < 0, we observe the following outcomes.

$$\begin{array}{c} P_{6}\left(x,t\right)=(\frac{\sqrt{C_{1}}\sqrt{C_{2}}\left(\lambda -2\mu \right)}{2\sqrt{3C_{3}^{2}(\lambda^{2}-4\mu )}}+\frac{b\sqrt{C_{1}}\sqrt{C_{2}}\left(-\lambda +\mu +1\right)}{\sqrt{3}\sqrt{C_{3}^{2}(\lambda^{2}-4\mu )}}V)e^{\left(\frac{itC_{1}C_{2}^{2}}{12C_{3}^{2}}\right)}, \end{array}$$ (21)

where

$$\begin{array}{c} V=\frac{(\lambda r_{1}-\sqrt{-\Theta }r_{2})\;\text{cos}\left(\frac{\sqrt{-\Theta }}{2b}\xi \right)+(\lambda r_{2}-\sqrt{-\Theta }r_{1})\;\text{sin}\left(\frac{\sqrt{-\Theta }}{2b}\xi \right)}{b(\left(\lambda -2\right)r_{1}-\sqrt{-\Theta }r_{2})\;\text{cos}\left(\frac{\sqrt{-\Theta }}{2b}\xi \right)+b(\left(\lambda -2\right)r_{2}+\sqrt{-\Theta }r_{1})\;\text{sin}\left(\frac{\sqrt{-\Theta }}{2b}\xi \right)}. \end{array}$$

Moreover, if $\left(\lambda -2\right)r_{2}+\sqrt{-\Theta }r_{1}=0$, then Eq (21) becomes

$$\begin{array}{c} P_{7}\left(x,t\right)=(\frac{\sqrt{C_{1}}\sqrt{C_{2}}\left(\lambda -2\mu \right)}{2\sqrt{3C_{3}^{2}(\lambda^{2}-4\mu )}}+\frac{b\sqrt{C_{1}}\sqrt{C_{2}}\left(-\lambda +\mu +1\right)}{\sqrt{3}\sqrt{C_{3}^{2}(\lambda^{2}-4\mu )}}V)e^{\left(\frac{itC_{1}C_{2}^{2}}{12C_{3}^{2}}\right)}, \end{array}$$ (22)

where

$$\begin{array}{c} V=\frac{\lambda -2\mu }{2b(\lambda -\mu -1)}+\frac{\sqrt{-\Theta }}{2b(\lambda -\mu -1)}\;\text{tan}\left(\frac{\sqrt{-\Theta }}{2b}\xi \right)). \end{array}$$

Moreover, if $\left(\lambda -2\right)r_{1}-\sqrt{-\Theta }r_{2}=0$, then Eq (21) becomes

$$\begin{array}{c} P_{8}\left(x,t\right)=(\frac{\sqrt{C_{1}}\sqrt{C_{2}}\left(\lambda -2\mu \right)}{2\sqrt{3C_{3}^{2}(\lambda^{2}-4\mu )}}+\frac{b\sqrt{C_{1}}\sqrt{C_{2}}\left(-\lambda +\mu +1\right)}{\sqrt{3}\sqrt{C_{3}^{2}(\lambda^{2}-4\mu )}}V)e^{\left(\frac{itC_{1}C_{2}^{2}}{12C_{3}^{2}}\right)}, \end{array}$$ (23)

where

$$\begin{array}{c} V=\frac{\lambda -2\mu }{2b(\lambda -\mu -1)}-\frac{\sqrt{-\Theta }}{2b(\lambda -\mu -1)}\;\text{cot}\left(\frac{\sqrt{-\Theta }}{2b}\xi \right). \end{array}$$ (24)

## Section 7: Graphical illustration

Graphs serve as powerful tools for conveying information and effectively illustrating the solutions to the problem in a logical manner. In this context, a series of graphs representing solutions for various parameter values were presented by using the SGEM and the $G^{\prime }/\left(bG^{\prime }+G+a\right)$-expansion method. The SGEM initially acts as a valuable instrument for revealing exact solutions across a wider range of nonlinear Schrödinger equations. Subsequently, we employ Mathematica 12 software to create visual representations of these exact solutions, specifically for both **SET 1** and **2**. Within these solutions, established through the SGEM, we encounter hyperbolic functions that encompass real and complex values. The outcomes identified in our analysis can be harnessed to assess real-world characteristics related to wave amplitudes and widths via these hyperbolic function solutions.

The hyperbolic function solutions offer valuable insights into critical parameters such as wavelength and frequency, as exemplified in the equations provided earlier. It’s worth noting that as wavelengths extend, their impact on a global scale becomes increasingly significant. Consequently, delving into the mathematical foundations of these natural phenomena becomes imperative, whether to mitigate their potentially destructive effects or harness them as valuable energy sources.

**Fig 1:**
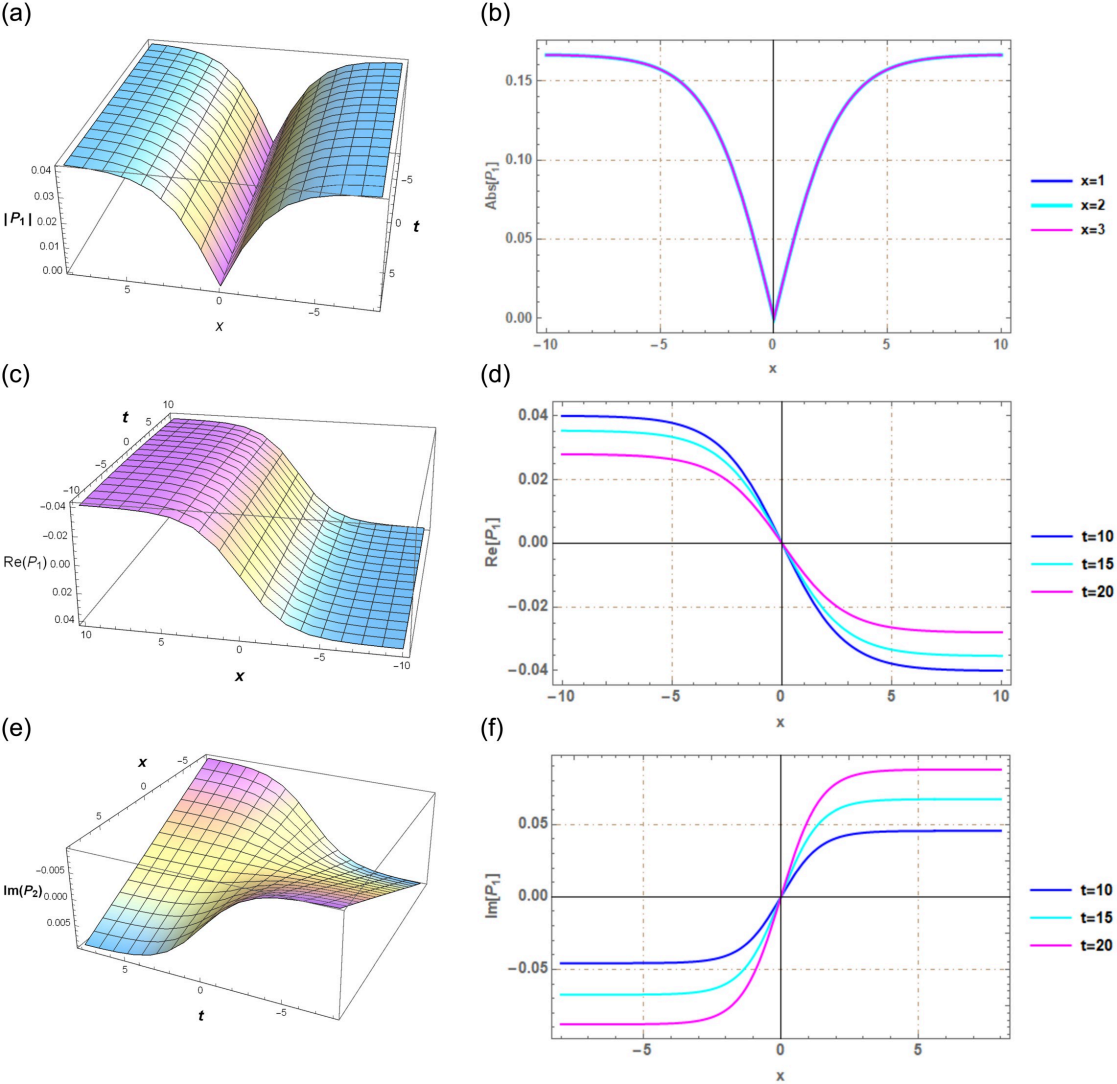
Solitary wave perspective via 3*D* and 2*D* portrayals of real and imaginary facets of the function *P*_1_(*x*, *t*) created using the SGEM.

**Fig 2:**
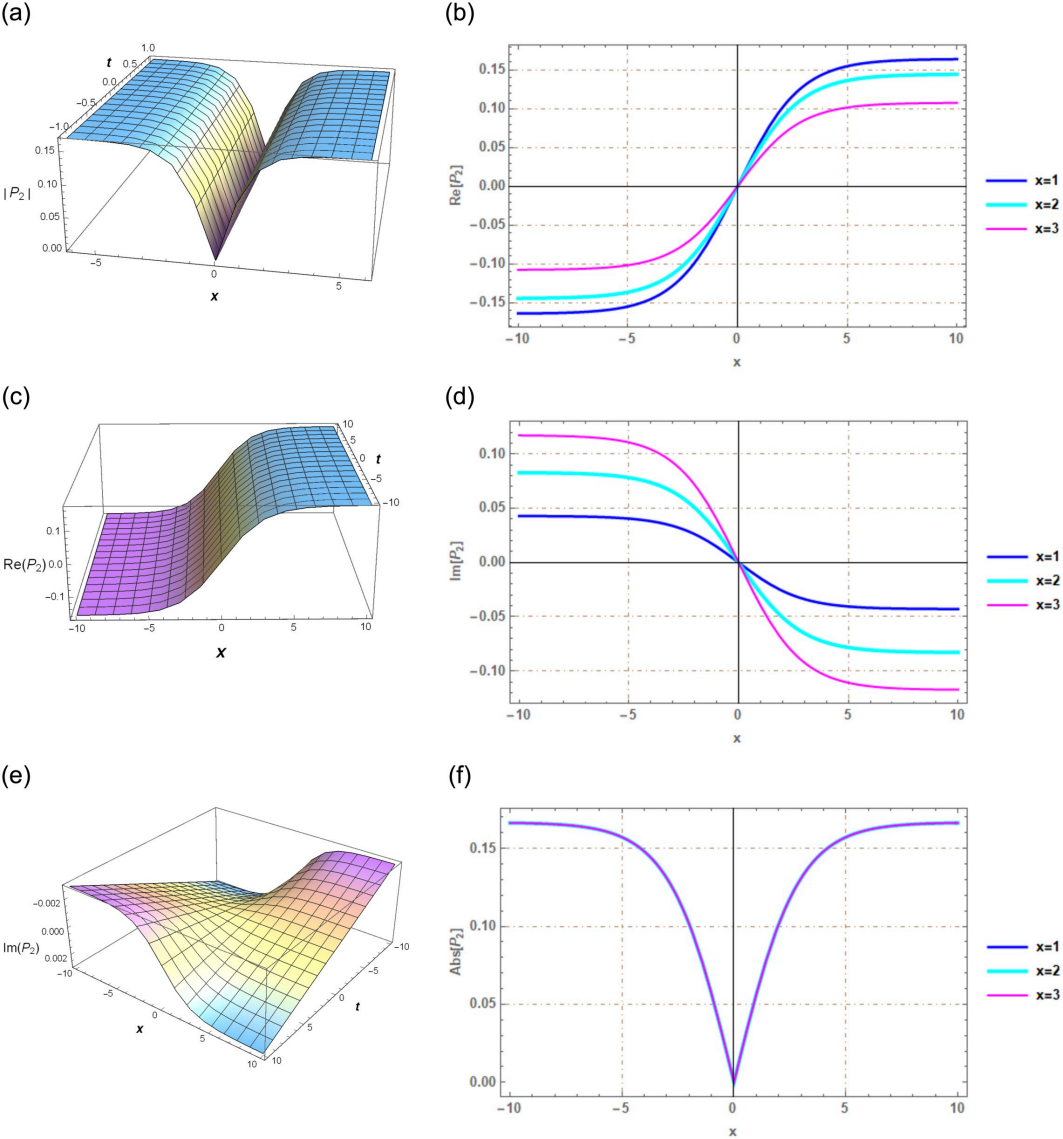
Solitary wave perspective via 3*D* and 2*D* portrayals of real and imaginary facets of the function *P*_2_(*x*, *t*) created using the SGEM.

Through the utilization of 3-D and 2-D graphs, this study effectively demonstrates the behavior of the real and imaginary components of the computed solutions, designated as *P*_1_(*x*, *t*) and *P*_2_(*x*, *t*). Specifically, the figures labeled as Figs 1 and 2 present the contextual values of the specified parameters: *A*_0_ = *B*_1_ = 0, *C*_2_ = 1, *C*_3_ = 4, and *C*_1_ = −1. Our study yields a diverse array of solutions, including Dark, Kink, anti-Kink, and single Soliton solutions. In the realm of optics, NLSE plays a pivotal role by elucidating the intricate behavior of optical pulses within nonlinear media. Soliton solutions, with their unique characteristics, particularly for nonlocal Schrödinger equations in optics, underscore the significance of our findings.

**Fig 3:**
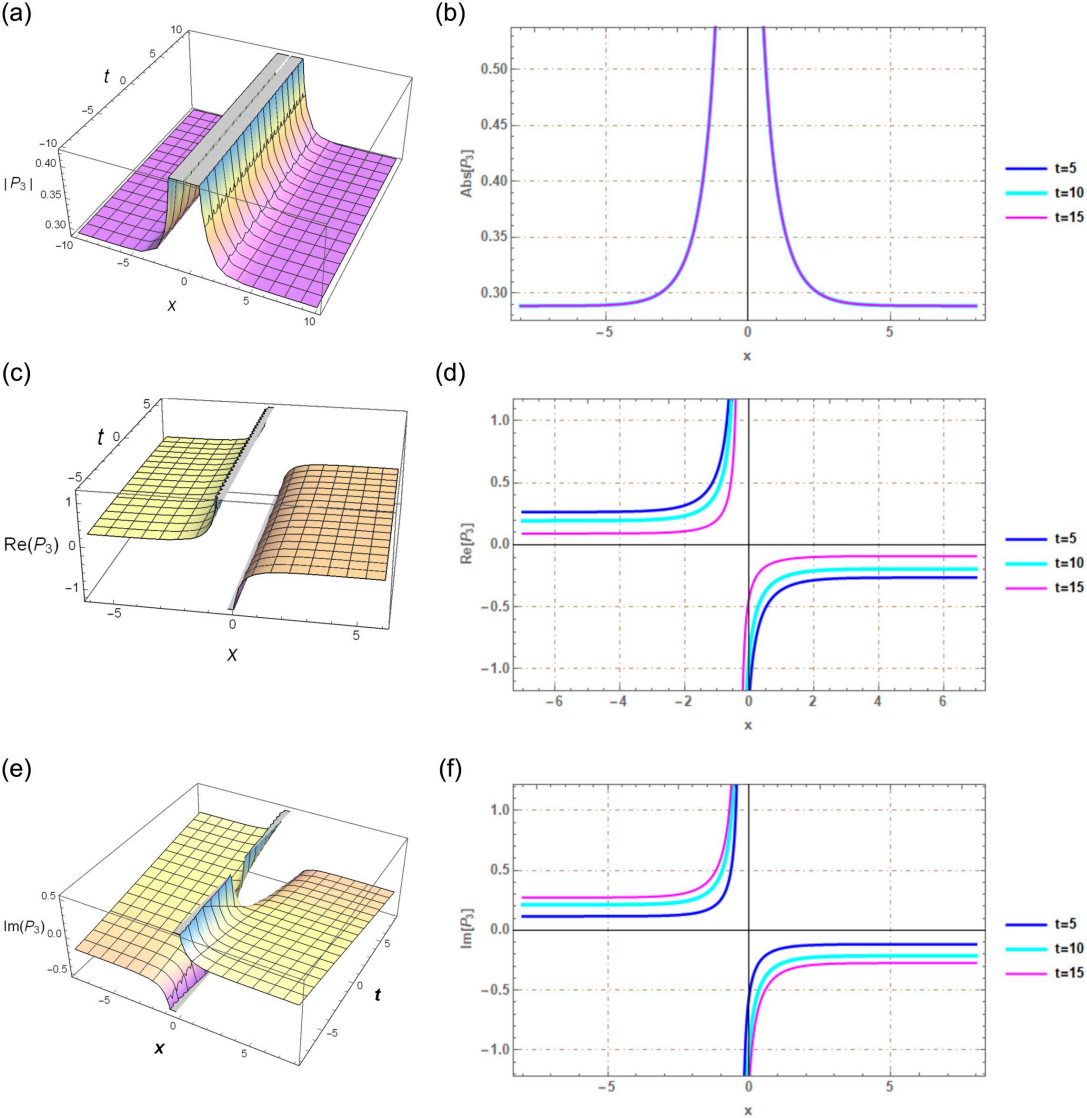
Solitary wave perspective via 3*D* and 2*D* portrayals of real and imaginary facets of the function *P*_3_(*x*, *t*) created using $G^{\prime }/\left(bG^{\prime }+G+a\right)$-expansion method.

**Fig 4:**
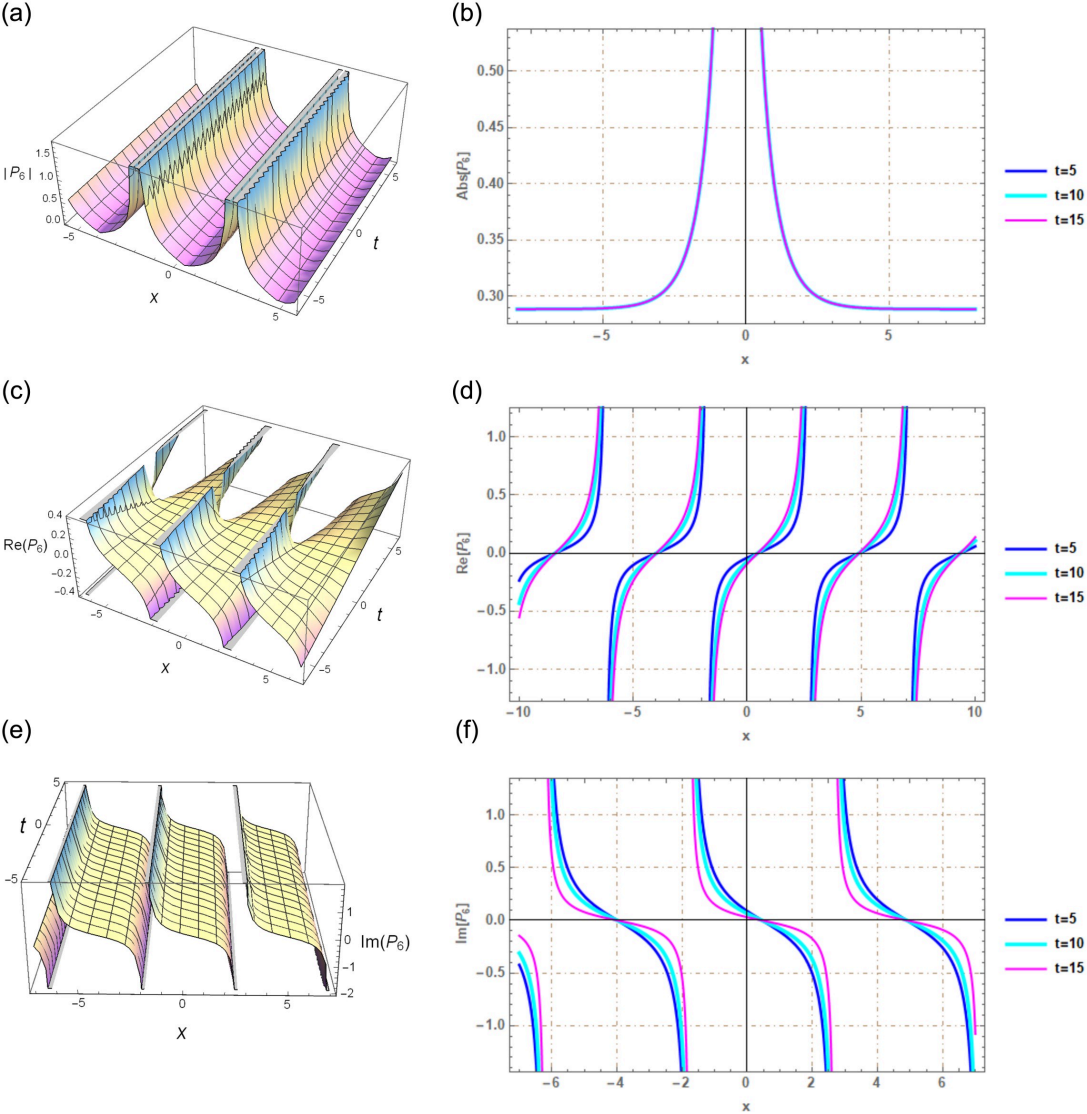
Solitary wave perspective via 3*D* and 2*D* portrayals of real and imaginary facets of the function *P*_6_(*x*, *t*) created using $G^{\prime }/\left(bG^{\prime }+G+a\right)$-expansion method.

The application of the $G^{\prime }/\left(bG^{\prime }+G+a\right)$-expansion method in our study proves to be an effective approach for discovering solitary wave solutions within a more extensive range of NLSEs. The graphical depictions notably illustrate these solutions’ solitary waveforms. Upon employing specific parameters, such as λ = 2.2, *μ* = 1, *r*_1_ = *r*_2_ = 1, *C*_1_ = *C*_2_ = 1, and *C*_3_ = −1, within our solution *P*_3_(*x*, *t*), as exemplified in Fig 3, novel soliton solutions are generated. Likewise, when we suggest values like λ = 1.2, *μ* = 1, *r*_1_ = *r*_2_ = 1, *C*_1_ = 1, *C*_2_ = 1, and *C*_3_ = 1 in our solution *P*_6_(*x*, *t*), illustrated in Fig 4, we obtain new solitary wave solutions. Utilizing Mathematica 12, we created graphical representations of the exact solutions mentioned above. These graphs encompass both real and complex solutions derived from the new soliton solution and are presented in both 3D and 2D formats.

## Section 8: Conclusion

This article focuses on exploring exact soliton solutions for the generalized nonlinear Schrödinger equation. The equation was characterized by higher-order dispersion with higher order nonlinearity, and a parameter associated with weak nonlocality. To address this challenging equation, we employed two well-established techniques: the sine-Gordon expansion method and the $G^{\prime }/\left(bG^{\prime }+G+a\right)$-expansion method. By applying these methods in conjunction with appropriate traveling wave transformations, we successfully derived novel and highly efficient solitary-wave solutions for this model. To gain insights into the equation’s physical implications, we harnessed Wolfram Mathematica 12 for generating both 3D and 2D graphical representations. These visualizations offer profound insights into various aspects of the equation’s behavior. Through manipulation of parameter values, we unveiled a variety of solutions, including kink-type, dark soliton, and solitary wave solutions. Our computational analyses underscore the effectiveness and versatility of these methods in addressing a wide range of nonlinear problems in the domains of mathematical science and engineering. Sometimes these techniques, may not be as reliable in producing accurate solutions due to their limited applicability to a diversity of equations and possible convergence problems. Future prospects involve the exploration of alternative analytical techniques, including the derivation of generalized solutions like Jacobi elliptic function solutions and multiple solutions. Furthermore, conducting bifurcation analysis, chaos analysis, and investigating modulation instability can be pursued. This research may lead to the discovery of N-soliton solutions, rogue waves, breathers, and various other intriguing solutions.
